# Exploration of validity evidence for core residency entrustable professional activities in Chinese pediatric residency

**DOI:** 10.3389/fmed.2023.1301356

**Published:** 2024-01-08

**Authors:** Shan Li, Xin Qi, Haichao Li, Wenjing Zhou, Zhehan Jiang, Jianguang Qi

**Affiliations:** ^1^Department of Paediatrics, Peking University First Hospital, Beijing, China; ^2^Department of Plastic Surgery and Burns, Peking University First Hospital, Beijing, China; ^3^Department of Respiratory and Critical Medicine, Peking University First Hospital, Beijing, China; ^4^School of Public Health, Peking University, Beijing, China; ^5^Institute of Medical Education and National Center for Health Professions Education Department, Peking University, Beijing, China

**Keywords:** competency, postgraduate medical education, entrustable professional activities, pediatrics, China, validity

## Abstract

**Introduction:**

This study seeks to explore validity and reliability evidence for core residency entrustable professional activities (CR-EPAs) that were developed by Peking University First Hospital (PKUFH) in 2020.

**Methods:**

A prospective cohort study was conducted in PKUFH. Trainers (raters) assessed pediatric residents on CR-EPAs over 1 academic year, bi-annually. Critical components within a validity evidence framework were examined: response process (rater perceptions), the internal structure (reliability and contributions of different variance sources), and consequences (potential use of a cutoff score).

**Results:**

In total, 37 residents were enrolled, and 111 and 99 trainers’ ratings were collected in Fall 2020 and Spring 2021, respectively. For rater perceptions, all the raters considered CR-EPAs highly operational and convenient. In all ratings, individual EPAs correlate with total EPA moderately, with Spearman correlation coefficients spanning from 0.805 to 0.919. EPA 2 (select and interpret the auxiliary examinations), EPA 5 (prepare and complete medical documents), EPA 6 (provide an oral presentation of a case or a clinical encounter), and EPA 7 (identify and manage the general clinical conditions) were EPAs correlated with other EPAs significantly. The results of the generalizability theory indicated that the variability due to residents is the highest (nearly 78.5%), leading to a large size of the reliability estimates. The matching results indicate that the lowest error locates at 5.933.

**Conclusion:**

The rating showed good validity and reliability. The ratings were reliable based on G-theory. CR-EPAs have a magnificent internal structure and have promising consequences. Our results indicate that CR-EPAs are a robust assessment tool in workplace-based training in a carefully designed setting.

## Introduction

Competency-based medical education (CBME) was widely used in postgraduate medical education (PGME) and has become a symbolic approach to reform medical education (1).

Competence is a complex, interrelated, multidimensional construct to be acquired by medical professionals for safe and effective clinical practice. By adhering to the two core principles of CBME, which involve redefining the criteria for a competent physician and emphasizing the achievement of competent graduates, CBME has demonstrated an enhancement in clinical practice and an improvement in patient safety (2). Given China’s large population, it has established a series of policies to ensure a safe health system with only qualified professionals practicing, emphasizing the implementation of CBME in PGME (3). In its capacity as a trailblazer in pediatric residency training within China, Peking University First Hospital (PKUFH) initiated its pediatrics residency program as far back as 1946. Remarkably, this initiative preceded the Chinese National standardized resident training guidelines by nearly seven decades (3).The pediatric residency program at PKUFH was accredited by the Chinese Medical Doctor’s Association (CMDA) and provisional accreditation by the Royal College of Physician and Surgeon of Canada (RCPSC). As proposed by Melle et al., we tried to implement five core components of CBME in the pediatric residency program (Appendix 1) (4) since 2012. The establishment of the Core Competency Framework for Residency Education by the China Consortium of Elite Teaching Hospitals in 2018 marked a significant milestone. Prior to this development, a comprehensive framework for residency education had not been in place (5).

The CanMEDS framework of RCPSC has been implemented in our institute as outcome competencies (Appendix 1). A stratified rotation is systematically conducted for each resident, wherein a highly specific competency-based assessment approach, encompassing both formative and summative evaluations, is meticulously delineated. Throughout the course of their rotations in various subspecialties, residents can expect to receive continuous feedback from their trainers and the program director, typically occurring at regular intervals of approximately every 2–3 months. The clinical competency committee (CCC) was held semi-annually. The rotation of certain subspecialties might be extended if a resident fails to meet the minimum criteria.

Through our practice, competency itself somehow is not easy to be understood nor to be observed by trainers, which creates a gap between competency and the daily tasks of doctors. As a tool to close the gap, the milestones initiative showed satisfactory validity as an assessment tool for competency (6); however, it is a rather complex system. Entrustable professional activities (EPAs), initiated by Ten Cate (7), provided a sound alternative approach for assessment in CBME (8–10), which might be the solution for sequenced progression of competency and programmatic assessment. EPAs are a bundle of clinical tasks vital to competency assessment (11, 12); in that way, EPAs can link routine clinical tasks to competency assessment. For instance, the commonplace task of “history taking” necessitates the demonstration of multiple competencies, encompassing roles such as medical expertise, effective communication, advocacy for health, and professionalism. Through the supervision of this “history-taking” process, trainers gain valuable insights into the trainee’s overall competency. After supervision, entrustment decision-making was performed accordingly so that the competency could be translated into trusted behavior, which is easier for implementation in a nationwide fashion.

Based on the foundation of CBME, we further began exploring the development of EPA in PGME. In 2020, a modified Delphi method consisted of two iterative rounds and one consensus meeting to develop the core residency entrustable professional activities (CR-EPAs; Figure A1 in Appendix 2). A 15-item competency assessment framework mainly focused on generic and core professional activities, as in Table 1 (13). The supervision rating scale of nine-point Likert items was initially set up according to Chen et al. in EPA implementation studies (14, 15). Considering that the nine-point span is too sparse to be informative in assessing the least requirement for residents (16), we modified the nine-point scale into an eight-point scale (Table 2). To minimize the need for faculty training, we use the same supervision scale level across all the CR-EPAs.

**Table 1:** CR-EPAs^a^.

EPA 1	Gather history and perform physical examination during patient encounter
EPA 2	Select and interpret the auxiliary examinations
EPA 3	Provide diagnosis and differential diagnosis
EPA 4	Develop the comprehensive management plan for patients
EPA 5	Prepare and complete medical documents
EPA 6	Provide oral presentation of a case or a clinical encounter
EPA 7	Identify and manage the general clinical conditions
EPA 8	Identify clinical emergency and critical illness and provide initial management
EPA 9	Transit and hand over the patient
EPA 10	Obtain informed consent for tests and/or procedures
EPA 11	Perform general procedures of a physician
EPA 12	Provide patient education and health advocacy
EPA 13	Deliver bad news to patients and/or family members
EPA 14	Deliver clinical teaching and instruct near-peers
EPA 15	Prepare and respond to public health events

^a^Peking University First Hospital; EPAs, entrustable professional activities; CR-EPAs (13), core residency EPAs.

**Table 2:** EPAs supervision scales as used in Peking University First Hospital Pediatric Department.

Description of competence	Original code (17)	Coded in this study
Trusted to observe only	1b	1
Trusted to practice EPA only under proactive full supervision as coactivity with rater	2a	2
Trusted to practice EPA only under proactive, full supervision with rater in room ready to step in as needed	2b	3
Trusted to practice EPA only under reactive/on-demand supervision with rater immediately available, all findings double checked	3a	4
Trusted to practice EPA only under reactive/on-demand supervision with rater immediately available, key findings double checked	3b	5
Trusted to practice EPA only under reactive/on-demand supervision with rater distantly available, findings reviewed	3c	6
Trusted to practice EPA unsupervised	4	7
Trusted to supervise others in practice of EPA	5	8

EPAs, entrustable professional activities.

The aforementioned CBME has been implanted in the PKUFH pediatric residency program. Aiming at understanding CR-EPAs in a practical assessment setting, revealing how it would integrate into our previous CBME system and explicit training perils and problems, we conducted a prospective cohort study in the pediatric residency training program of PKUFH. This research aimed to gather validity evidence for CR-EPAs in the pediatric residency training setting and inspect if CR-EPAs could provide reliable and meaningful data for evaluating residents. The study was organized into a three-component investigation: response process (rater perception), internal structure (variance components reliability), and consequences (potential use of a cutoff score).

## Materials and methods

### Study design

This study aimed to gather validity evidence for CR-EPAs. To gather information ahead and follow the trainees for a period of time, a prospective cohort study that implemented CR-EPAs in the PKUFH pediatric residency training program over 1 academic year (July 2020 to June 2021) was conducted. The rating was achieved in January 2021 and July 2021, denoting residents’ performance in Fall 2020 and Spring 2021, respectively.

The SPSS (version 23.0.0) and R (version 4.0.1) were used for statistical analysis. The Prism (version 9.0.0) was used for visualizing the analysis.

### Ethics approval and consent to participate

In accordance with the Declaration of Helsinki, the institutional review board at PKUFH granted this study (2021-107). Before participating, informed written consent were obtained from each resident and trainer in accordance with relevant guidelines and regulations.

### Study participants

As forementioned well-launched CBME in PKUFH Department of Pediatrics, both the trainers and residents were familiar with the concept of competency and competency assessment. Residents in the PKUFH Department of Pediatrics residency training program were selected as the subjects of this study. It would maximumly reduce systemic errors to a certain extent. Inclusion criteria were (a) having finished at least 9 months of rotation during the study period and (b) being willing to be assessed by trainers. Residents were divided into different postgraduate year (PGY) groups according to their rotation year in July 2020. The demographic information was collected.

### Response process

Trainers rated each resident according to their previous rotated subspecialties. These trainers were, therefore, the raters throughout this article. A series of tutorials on the EPAs’ concept was delivered to the trainers to minimize the inter-rater difference.

The rating processes were performed through an online survey platform (http://www.wjx.cn/). The trainers could complete the ratings by either computer or mobile devices. Each form contained 15 items of CR-EPAs (Appendix 3). A link containing CR-EPA supervision rating and the list of trainees to be assessed was sent to each trainer every 6 months. For each trainee, multiple times of ratings would be conducted by different trainers according to their previous rotation and performance. To avoid missing data, the survey could only be submitted after all EPAs of a target trainee were fully observed. The raters were allowed to select “unable to rate” certain EPAs if they felt inadequate supervision/observation opportunities or insufficient qualifications. The time consumed for each rating was automatically recorded. For each resident, overall performance was the average of multiple trainers’ ratings.

After completing the rating, we compared EPAs across PGYs and assessment periods. A focus interview with eight raters was conducted to document comments and thoughts about using CR-EPAs in practice, ensuring the completeness of the response process investigation. All interviewees’ questions were open-ended: “how do you feel when you were using CR-EPAs in practice.”

### Internal structure

The internal structure was investigated through (1) associations between any pairs of EPAs via Spearman correlation analysis, (2) discrimination of each EPA via Spearman correlation analysis, and (3) variance and reliability estimation via generalizability theory (G-theory) (18). Specifically, G-theory was used to decompose variance components of the assessment, and the estimation was achieved by using restricted maximum likelihood (REML) (19). As psychometrics theory indicates, the correlation between items (e.g., EPAs in our context) provides evidence for validity: they are expected to have a moderate-to-high correlation to show a good measurement structure. The correlation between an item (again an EPA in our context) and its sum/mean (as an overall performance for the entire assessment) is essentially “discrimination.”

Many performance-based assessments are investigated through Cronbach’s α, inter-rater reliability, inter-rater agreement, or concordance that all belong to classical test theory (CTT) (20–22). This study, however, utilized G-theory as it is more proper for the setting of our CR-EPAs. The reasons for using G-theory instead of others are listed below:

1. Instigating qualities of education assessment (i.e., validity) also most always involves measurement theories and their quantifying frameworks, including CTT, G-theory, and item response theory (23). Therefore, G-theory is a candidate for the study.
2. Compared with CTT that simply assumes that observed performance consists of true ability effect and error effect (i.e., the well-known *X* = *T* + *E* and each effect correspond to variance such that $\sigma^{2}\left(X\right)=\sigma^{2}\left(T\right)+\sigma^{2}\left(E\right)$ ), G-theory is compatible with designs with *multiple facets* such as raters, items, groups, and occasions (24, 25), each of which is an effect affecting the observed scores. For instance, in performance assessment where a is the *p* × *i* × *r* design present (each person *p* is graded by every rater *r* on each task/item *i*), G-theory can decompose observed response data as $X_{pri}=\mu +v_{p}+v_{i}+v_{r}+v_{pi}+v_{ir}+v_{pr}+\epsilon_{pri}$ , where an observed score, $X_{pri}$ , for person *p* on item *i* rated by rater *r* is made of the grand mean μ, person effect $v_{p}$ , item effect $v_{i}$ , rater effect $v_{r}$ , interaction terms of any two random effects, and error effect $\epsilon_{pri}$ . Each of these effects involves variance as CTT does, and their values can indicate the proportion of an effect contributing to the data. To illustrate, the proportion of rater and item effects count for 80 and 10% of the total variance of the data and then intuitively one would consider the inconsistency between raters is high, while the items are more homogenous.
3. IRT is used more in large-scale standardized (multiple-choice) assessment (26), where the sample sizes are generally large. In certain simplified scenarios, such as scoring with the rating scale, IRT as G-theory can be used interchangeably (27). However, when *multiple facets* are available and non-large-scale scenarios are present, G-theory makes a more appropriate and reliable choice, especially when the designs are complex such as random-distributed and/or nested structures (28).
4. Competence/performance-related investigations through G-theory in the field of medical education have been seen more in the literature (24, 25) also conveying that our methodological adoption is a strong fit for the present study, which involves different EPAs, raters, and randomly crossed structure between raters and residents.

Variance estimates of G-theory allow calculating the level of (1) dependability (criterion- or domain-referenced) and (2) generalizability (e.g., norm-referenced interpretations of test scores), which are akin to reliability in CTT. G-theory enables researchers to make decisions on how to alter the reliability coefficient to a specific level. For instance, if G-theory shows a large variance in the rater effect implying a lack of consistency among themselves, a decision study (namely, D-study) will be informative to the prediction: how many raters are demanded to reach a specific coefficient level. In our study, the effects of raters, items (i.e., EPAs), residents, and their interactions were considered. Their estimation was achieved via the R software (29).

### Consequence

Finally, consequence analysis was defined to investigate the potential use of EPA scores in future competency screening. In practice, administrators and raters tend to use an observed (mean or sum) score to evaluate if a resident meets the minimal requirements of the competency assessment. This implementation involves setting a cutoff score that theoretically consists of the least measurement errors or makes the highest sense through scientific reasoning. In this study, we aligned the observed (mean) EPA scores of each resident with true scores (i.e., the ability estimates from G-theory modeling after excluding other noises such as rater effect and item effect), and the scores’ uncertainty/errors yielded from the aforementioned G-theory analysis. Ideally, the cutoff observed score should correspond to the true score level with the lowest uncertainty/errors, namely, the most reliable threshold setting from a data-driven perspective (30).

## Results

### Descriptive statistics

Thirty-eight pediatric residents were enrolled in this study; one was excluded due to incomplete rotation. The demographics of 37 residents are shown in Table 3. In total, 23 raters (trainers) participated in the assessment, and their demographics are shown in Table 4.

**Table 3:** Demographic of 37 participating pediatric residents.

Demographic	No. (%), except where noted
*Gender*	
Female	10 (27)
Male	27 (73)
Age, average ± SD	26.8 ± 3.1
*Level of training*	
PGY-1 resident	13 (35)
PGY-2 resident	11 (30)
PGY-3 resident	13 (35)
Number of ratings of each resident in fall 2020, average ± SD	3.0 ± 1.2
1	3 (8)
2	12 (32)
3	9 (24)
4	8 (22)
5	5 (14)
Number of ratings of each resident in in spring 2021, average ± SD	2.7 ± 0.7
1	2 (5)
2	12 (33)
3	19 (51)
4	4 (11)

PGY, postgraduate year.

**Table 4:** Dermographics of 23 trainers (raters).

Demographic	No. (%), except where noted
*Gender*	
Female	18 (78.2)
Male	5 (21.8)
Age, median (range)	33 (29–47)
*Degree*	
Medical doctors	21 (91.3)
Master’s degree	3 (8.7)
*Year enagaged in teaching*	
<1 year	4 (17.5)
1 to <2 years	7 (30.4)
2 to <3 years	5 (21.7)
≥3 years	7 (30.4)
*Subspecialty*	
General pediatric	1 (4.3)
Respiratory	2 (8.6)
Pediatric intensive care	2 (8.6)
Neurology	4 (17.2)
Nephrology	3 (13.0)
Cardiology	2 (8.6)
Neonatology	4 (17.2)
Hematology	2 (8.6)
Neonatal intensive care	3 (13.0)

**Figure 1:**
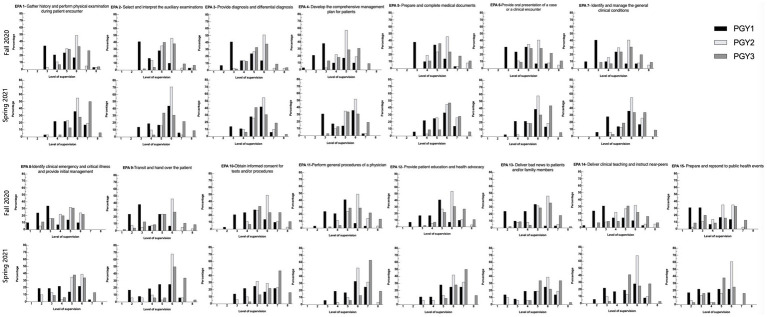
Distribution of the level of supervision assignments by Clinical Competency Committees for each of the CR-EPAs (see Table 1) using the scales (see Table 2). In each panel, bars represent the percentage of ratings for each level of supervision by postgraduate year (PGY). (Black bars= PGY1; Light gray bars= PGY2, dark gray bars= PGY3). For each EPA, data from July 2021 are displayed below those from Jan 2021.

In total, 111 and 99 ratings were received for the two investigation periods, respectively. Each resident received 3.0 ± 1.2 ratings in Fall 2020 and 2.7 ± 0.7 in Spring 2021 from trainers, respectively. Since each EPA needs to be completed before submission, no data for the EPA assessment was missing. The supervision rating results of each EPA through PGY1 to PGY3 in Fall 2020 and Spring 2021 are shown in Figure 1.

### Response process

All the raters (trainers) fulfilled the supervision rating within 2 weeks of the assignment. None reported “unable to rate.” The rating data were analyzed, and the results were reviewed with the raters (Figure A2 in Appendix 2). The emergent theme of both strengths and limitations was listed during the focus interview in Table 5. All the raters considered CR-EPAs to be highly operational and convenient. The conclusion is consistent with the time consumed to complete one rating—the minimum time is 37 s, while the maximum is 1700 s, with a median of 143 s. The raters believed that the proposed CR-EPA supervision rating was consistent with their clinical observations of a specific resident and a realistic reflection of the resident’s clinical competence. If using traditional assessment tools (such as 360° assessment), raters often overestimate residents’ “actual behavior” and cannot genuinely differentiate students’ levels. However, it would be more mutual and objective when using supervision decision-making as the measurement scale, thus achieving good consistency among different raters.

**Table 5:** Aspects of the CR-EPAs supervision decision-making.

Emergent theme	Description
*Favorable aspects*	
Characteristics of CR-EPAs	CR-EPAs covered major generic clinical behavior and was a clinical-based, bed-side based assessment. Raters could easily make a supervision decision based on residents’ clinical behavior, and CR-EPAs supervision level could reflect residents ‘actual clinical competency.
Comparing to other assessment tools	CR-EPAs was a more mutual and objective assessment tool, compared with 360° assessment, and is clinical-based assessment compared with traditional structural case-interview. CR-EPAs illustrated more clinical competencies compared with mini-CEX and DOPS.
*Areas to improve*	
Assessment Platform	CR-EPAs should become a regular assessment with an interval of 2 to 3 months based on the rotation of residents. The assessment should have a specified electronic platform which is easy to review the previous results both for residents and raters and should have longitudinal data for certain residents and raters.
Lack of discriminations between PGY2 and PGY3 residents	Data were lack of discriminations between PGY residents. CR-EPAs was a generic clinical behavior. PGY-2 and PGY-3 residents shared similar responsibilities. The growth trajectory would be flat in the next 2 years. EPAs based on general pediatric training should be developed in future to fulfill the gap.

### Internal structure

**Figure 2:**
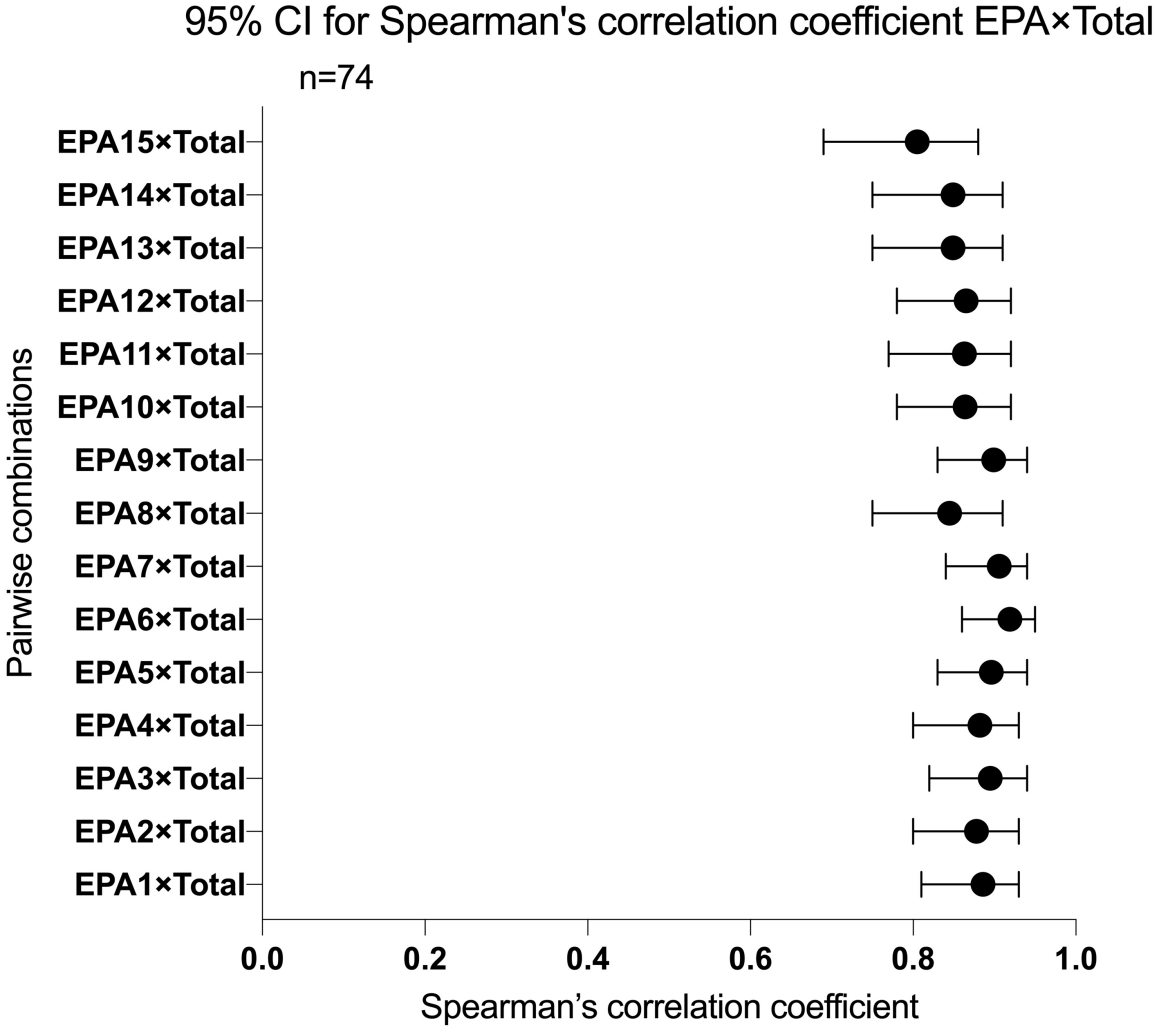
95% CI for Spearman’s correlation coefficient EPA items with total score.

In all ratings, individual EPAs correlate with total EPA moderately, with Spearman correlation coefficients spanning from 0.805 to 0.919, recorded in Figure 2, indicating that, overall, items nested within the target assessment possess good power in distinguishing residents’ competency. Spearman correlation coefficient between all EPA pairs ranged from 0.541 to 0.926, with a median of 0.759 (Q1 0.697, Q3 0.827), recorded in Table A1 in Appendix 2. None of the EPA pairs’ correlation was below 0.3. In total, 20 out of 105 (19%) individual EPA pairs’ correlation coefficient was above 0.85. Among those EPA pairs, EPA 2 (select and interpret the auxiliary examinations), EPA 7 (identify and manage the general clinical conditions), EPA 6 (provide an oral presentation of a case or a clinical encounter), and EPA 5 (prepare and complete medical documents) were the ones that significantly correlated with other counterparts.

The results of generalizability theory modeling were presented in Table 6, showing the estimates of the variance components for residents, raters, EPAs, the interactions between residents and EPAs, the interactions between raters and EPAs, the interactions between raters and EPAs, as well as the residuals. Variability due to residents is the highest (nearly 78.5%), leading to a large size of the reliability estimates (i.e., G-Coefficient and Φ-Coefficient estimates are both higher than 0.75). Two interaction terms—the one between residents and EPAs and the other between raters and residents—are lower than 1%, implying that these “noisy signals” are barely influential to the assessment. On the other hand, the interaction between raters and residents, although not high in a relative sense, shows the total variability related to raters is 8.4% (i.e., 0.099, 0.130, and 0.005). The contributions from EPAs are low and indicate a high consistency between the item levels.

**Table 6:** Variance decomposition and reliability estimates via generalizability theory.

Effects	Variance component (VC)	% VC	*df*	G-Coefficient	Φ-Coefficient
Resident	0.925	78.5	1	0.871	0.785
Rater	0.099	8.4	3		
EPA	0.013	1.1	15		
Resident:EPA	0.000	0	15		
Rater:EPA	0.005	0.4	45		
Rater:Resident	0.130	11	3		
Residual	0.007	0.6	45		

### Consequences

**Figure 3:**
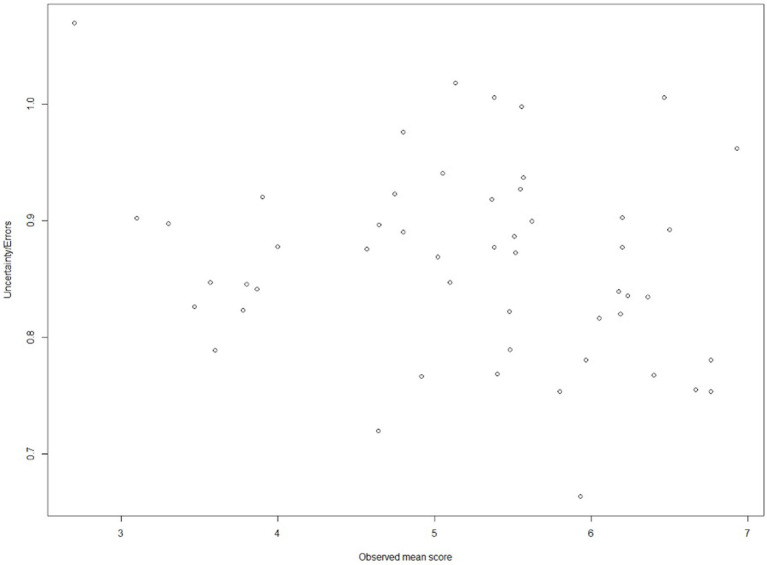
Relation between observed mean scores and corresponding uncertainty in a cut-off setting.

At each given observed mean score (i.e., overall performance), bootstrapping the G-theory yielded a set of true score estimates allowing the construction of an uncertainty range, which reflects the estimation precision. The matching results are contained in Figure 3, indicating that the lowest error is located at 5.933. On the other hand, when the overall performance is 2.700, the errors become the largest—1.069.

## Discussion

To the best of our knowledge, this is the first study in China to implement EPAs in the pediatric residency program. In our study, rating data sets on CR-EPAs during 1 academic year were employed to verify their reliability and validity, emphasizing the educational and clinical outcome of CR-EPA-based assessment in China’s 3-year standardized pediatric residency training.

Competency assessments require good reliability. The generalizability coefficients of CR-EPAs are 0.871, the Φ-coefficient of CR-EPAs is 0.785, and both coefficients are above 0.75, which indicates that the use of instruments in the given context is reliable. In comparison with other assessments of this kind, the reliability estimates are sufficiently high for performance-based tasks. For example, Meyer et al. (31) showed that the generalizability coefficients of a pilot preclinical entrustment assessment in undergraduate medical education fall between 0.66 and 0.52. The coefficients are decomposed into different sources, of which the resident effect dominates the variance. Surprisingly, the rater and the rater-related interaction effects consume a larger partition than others, which is usual because ratings are too subjective to be highly consistent among all raters. However, the proportions of the rater and the rater-related effects are still low, reflecting positive signs of the series of rater training prior to the present study.

Supervision decision-making is widely used in pediatrics throughout the world (32). PKUFH Pediatrics Department has a long history of CBME. Hence, reliability could reach such a high level. Quoted for the focus review, supervisors describe CR-EPAs as a clinical-based, bed-side-based assessment tool, and supervisors could easily make a supervision decision based on clinical behavior. CR-EPAs were more reciprocal and objective when using supervision decision-making as the measurement scale, thus achieving good consistency among different raters.

In this study, CR-EPAs also showed relatively good validity evidence. The assessments made in Spring 2021 were higher than those made in Fall 2020 in most EPAs (Figure A2 in Appendix 2). These results were consistent with the expectation that residents require less supervision as their skills improve with more experience and teaching. The developmental trajectories are evident in aggregate data, and similar results were found in other studies based on pediatric fellows. However, there was no upward trajectory from Fall to Spring for EPA 15 (apply and respond to public health events) possibly due to a lack of practice and observation. It is a common problem the world is facing in the post-pandemic era. A specialized training course should be implemented to enhance the training. As for the internal structure validity evidence, individual EPAs moderately correlate with the EPA total score, indicating that items nested within the target assessment possess good power in distinguishing residents’ competency. As for the inter-EPA correlation analysis, most EPA pairs were moderately correlated, while a few EPAs were significantly correlated with each other. Those EPAs are EPA 2 (select and interpret the auxiliary examinations), EPA 5 (prepare and complete medical documents), EPA 6 (provide an oral presentation of a case or a clinical encounter), and EPA 7 (identify and manage the general clinical conditions). Those EPAs were the most fundamental meta-EPAs and correlated with other EPAs significantly. Those EPAs included common clinical scenarios were that more observable for supervisors and, hence, more comfortable in judgment.

Although serving as an initial exploration in the present study, the consequence part provides insights into the decision-making use of EPAs. The data-driven result shows that supervision Level 6 (5.933) nearly corresponds to the lowest error for assigning residents to binary classes, which are conventionally interpreted as “pass/fail,” “competent/incompetent,” and “qualified/unqualified.” The cutoff score is well aligned with Chen (17) who claims that level 6 (Chen’s Level 3c) should be the threshold when residents graduate from a program (the end of PGY-3 training). Hence, from the perspective of G-theory, Level 6 should be the cutoff value for supervision levels of CR-EPAs for residents.

### Limitations

Our study has several limitations. It is a single-center, small sample-size study. The supervision rating timespan was half a year. It would be less likely to reflect a real-time improvement in the residents. The study was conducted only within 1 academic year in a single center. Furthermore, as the lack of an electronic platform specified for CR-EPAs ratings, our ratings were conducted on a survey platform, and all the EPAs were listed in a single survey; This may lead to a halo effect. Fortunately, an e-portfolio specified for CR-EPAs ratings will launch in our center soon, which can solve the problem in future.

## Conclusion

We developed an eight-level supervision scale for CR-EPAs and implemented it in a pediatric residency training program of PKUFH. The ratings were reliable based on G-theory. CR-EPAs have a fine internal structure and the consequences of using them for binary decision shows reasonable utility. Our results indicate that CR-EPAs can serve as a robust assessment tool in workplace-based training in a carefully designed setting.

## Data availability statement

The raw data supporting the conclusions of this article will be made available by the authors, without undue reservation.

## Ethics statement

The studies involving humans were approved by the institutional review board at Peking University First Hospital. The studies were conducted in accordance with the local legislation and institutional requirements. The participants provided their written informed consent to participate in this study.

## Author contributions

SL: Formal analysis, Investigation, Project administration, Writing – original draft, Software. XQ: Conceptualization, Funding acquisition, Methodology, Supervision, Writing – review & editing. HL: Conceptualization, Resources, Visualization, Writing – review & editing. WZ: Formal analysis, Validation, Writing – original draft. ZJ: Data curation, Formal analysis, Methodology, Validation, Writing – review & editing. JQ: Conceptualization, Funding acquisition, Investigation, Project administration, Resources, Visualization, Writing – review & editing.
