# Supplementary material for: Exploration of validity evidence for core residency entrustable professional activities in Chinese pediatric residency
Source: Front Med (Lausanne). 2024 Jan 8;10:1301356. doi: 10.3389/fmed.2023.1301356 (PMC10801054; doi:10.3389/fmed.2023.1301356)
Supplement: Supplementary file 1 [file Data_Sheet_1.zip › Data_Sheet_1/Appendix_1.pdf]

Appendix 1

Brief Introduction for Pediatric Residency Program in Peking University First Hospital

As the pioneers of pediatric residency training in China, Peking University First Hospital (PKUFH) established pediatrics residency training system in 1946. In 2012, a graduate medical education cooperation center between PKUFH and Royal College of Physician and Surgeon of Canada (RCPSC) was established. Since then, competency based medical education was introduced in the Pediatric Department of PKUFH, and a comprehensive formative assessment system was formed. The following is the brief introduction for Pediatric Residency Program in PKUFH.

ADMINISTRATIVE STRUCTURE

We established Residency Program Committee since June 2012, in the assistance of the planning、organization and supervision for the Residency Program. The Residency Program Committee constitute chairman （director of Pediatric department）、director （vice-director of Pediatric department in charge of medical education）、teaching secretary/assistant、managers in rotation sites and representatives of residents. The representatives of residents are elected annually (Figure 1). The residency program committee meeting is held every three months, the common problems that residents are facing, and the specific solutions are discussed during the committee meeting, and meeting records were documented. Besides quarterly committee meetings, the committee keeps in touch with members and residents via group discussion、telephone、e-mail, etc. Hence problems would be easily found during implementation of program and solutions would be discussed immediately.

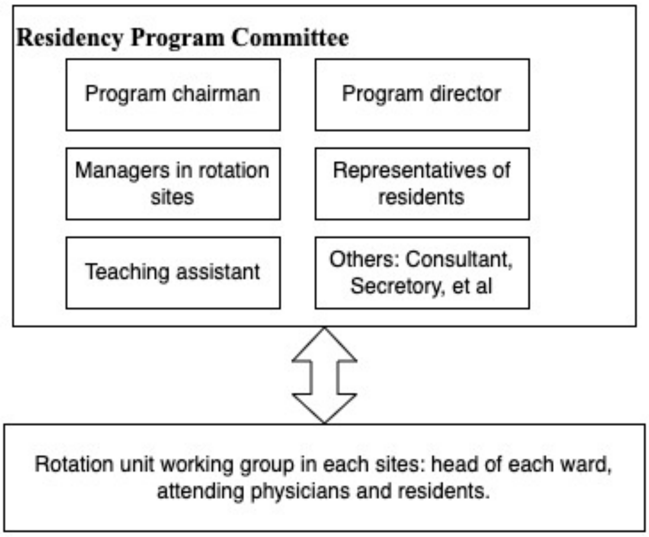

Appendix 1 Figure 1 Residency Program Committee structure

GOALS AND OBJECTIVES

After the training, residents should be a specialist acknowledge of child normal development and general diseases encountered in infants, children, and youth. Residents must demonstrate the requisite knowledge, skills, and attitudes for effective patient-centered care and service to a diverse population. After training, residents should be able to be a pediatric consultation, who know the basic medical sciences and latest research findings and could make independent clinical decisions. The general content of the training will include experience and study in the comprehensive care of children with physical and psychosocial challenges. The resident will learn the skills to work collaboratively and to provide consultation to other medical and health disciplines dealing with infants and children, especially with Psychiatry, Surgery and Obstetrics. The resident will acquire the professional attitudes to work with other health disciplines in a variety of health care service models. The resident will develop the skills of a self-directed life-long learning. The resident will learn the skills to critically appraise both his/her practice as well as the practice of Pediatrics. At the completion of training, the resident will have acquired the following competencies and will function effectively as a medical expert、communicator、collaborator、manager、health advocate、scholar and professional.

STRUCTURE AND ORGANIZATIN OF THE PROGRAM

The rotation and other educational experiences are organized well, and each resident can fulfill the educational requirements and achieve competence in the specialty or subspecialty. We provide all the components of training outlined in the specialty-specific documents, including classes, discussions, and lectures. The residents are appropriately supervised according to their level of training, ability, and experience. Their responsibilities are gradually increased according to program year of training, and experience. Besides clinical duty, senior residents should be responsible for the guidance and tutor of junior residents and interns. The on-call duties are assigned, and specific disease and procedures required are listed clearly. The residents should take charge of at least 3 to 5 patients at the same time.

According to the syllabus of standard residency training in Beijing, pediatric residency training needs 3 years of rotation. Mandatory training includes respiratory, hematology, nephrology, neurology, neonatology, cardiology, gastroenterology, ICU, infectious department, outpatient and emergency care (3 month for each unit). And preventive medicine requires 1-2 months, paramedical unit 1-2 months. Elective training includes endocrinology、rheumatology and inherited metabolic ward，residents can choose one.

A stratified rotation is performed (Figure 2): the rotation in each inpatient unit is split into two parts-2 months in the first round, and 1 month in the second round. Meanwhile residents would require more clinical responsibilities in the second round.

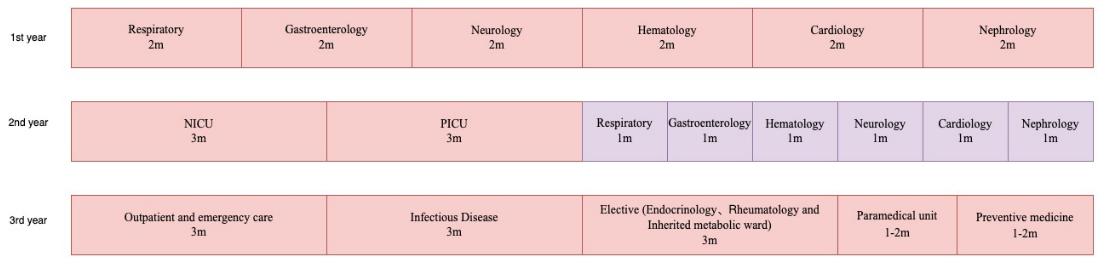

Appendix 1 Figure 2 Resident rotation arrangement

RESOURCES

As one of the famous tertiary hospitals in China, we have a sufficient number and variety of patients. The total clinical visits are about 250,000 per year, and the in-patient number is about 14,000. We have plenty of subspecialties, including cardiology、hematology、rheumatology、gastroenterology 、inherited metabolic ward、hematology、infectious ward、nephrology、neurology、pulmonology、developmental pediatrics and clinical immunology, which can meet the educational demand of the residents.

We have enough qualified teaching staff from a variety of medical subspecialties and other health professions to provide appropriate teaching and supervision of residents. We have 56 teachers in the program, among which there are 24 senior professionals, 15 sub-senior professionals, 17 attending doctors and all the teachers are certificated by the higher education authorities. Each resident has a tutor during the rotation, who is associate or chief doctor. The tutors are asked to keep in touch with residents, and offer help in clinical work、research、daily life and mental status.

CLINICAL, ACADEMIC, AND SCHOLARLY CONTENT OF THE PROGRAM

The clinical, academic, and scholarly content of the program must be appropriate for university postgraduate education and adequately prepare residents to fulfil all the CanMEDS Roles of the specialist. The quality of scholarship in the program will be demonstrated by providing opportunities for residents to get in touch with patients as much as possible, such as clinical discussions at the bedside, in clinics, in the community, and in seminars, rounds, conferences and so on. The clinical teaching activities focusing on CanMEDs are listed in Table 1.

Appendix 1 Table 1 CanMEDS Competency teaching in the clinical and non-clinical setting

CanMEDS Competency	Teaching in the Clinical Setting	Teaching in the Non-clinical Setting
**Medical Expert**		
Clinical work	Responsible for the patients<br>3-level ward round system	Courses on the knowledge, clinical skills, and clinical progress
**Communicator**		
written communication	Revision of medical records<br>Check and feedback of contents of medical records	Training for medical files writing
oral communication	Medical history reporting, shift and ward round	Communication courses<br>Specific communication training (case study, role play)

CanMEDS Competency	Teaching in the Clinical Setting	Teaching in the Non-clinical Setting
**Collaborator**		
participate effectively and appropriately in an interprofessional healthcare team	Grand rounds, consultation and referral as well as accompany the patient to take an examination	CPR drill, community practise, situational teaching
effectively manage conflict	Routine medical treatment, duty, and emergency treatment in emergency rotation	Lectures<br>Situational teaching
**Leadership**		
management, administration, and leadership	Examination of medical record (clinical track)<br>Experience the management of medical quality	Lectures (related humanistic lecture)<br>Situational teaching
management of practice	Ward rounds	literature reading (evidence based medicine)<br>Team based teaching<br>Journal-club
allocation of health care resources	Clinical diagnosis and treatment guidelines	Rotation in administrative department
QA/QI activities	Various medical treatment activities	Lectures：<br>Management of medical quality<br>Rotation in administrative department：<br>Management of medical quality
**Health Advocate**		
advocate for the health of communities they serve	Health education	Community propaganda<br>General medical treatment
teaching of diversity		
**Scholar**		
teaching of other residents, medical students, patients, families, the public and other health professionals	Clinical teaching	Teaching skills<br>Small lectures, literature reviews
critical appraisal, biostatistics relevant to the interpretation of the medical literature, and apply this appropriately to practice decisions	Teaching rounds (with case related literature review)	Journal club<br>Case discussion
self-assessment and self-directed life-long learning		Journal club
conduct scholarly project, participate in research	Joint case discussion<br>Discussion of difficult cases and consultation crossing division	Scientific exchanges, project opening report<br>Participate in research projects<br>Participate in academic conferences and

CanMEDS Competency	Teaching in the Clinical Setting	Teaching in the Non-clinical Setting
		communications
**Professional**		
professional conduct and ethical behaviours	Routine clinical activity<br>Discussion of difficult cases and consultation	Teaching salon, courses<br>Situational teaching
specialty-specific biomedical ethics		Lecture and simulated training:<br>Situational case teaching
set priorities and manage time to balance patient care, practice requirements, outside activities, & personal life,	Routine clinical activity	Lecture and simulated training:<br>Situational case teaching
deal with medical errors/adverse advents	Routine clinical activity	Courses<br>Rotation in administrative department, medical quality improvement
medical legal issues	Routine clinical activity	courses or lectures

ASSESSMENT OF RESIDENT PERFORMANCE

Both formative and summative assessment are used. Summative assessments include national and territory exams of MCQ and OSCE. Formative assessments are critical to residency training. We provide residents with kinds of formative assessments include: mini-CEX、 DOPS、SOAP case report and 360 degree assessment. Clinical competency committee（CCC）was held semi-yearly, and rankings were made for each residents.

During the rotation of subspecialties, residents will receive persistent oral feedback from senior physicians. In the medium of rotation, they will receive feedback of the results of mini-CEX、DOPS through a face-to-face meeting. After finishing the rotation, other faculties (eg. other residents and nurses) and patients or their family give a 360-degree assessment to the resident, which help to evaluating the attitudes professionalism, communication abilities and teaching abilities. The results of all the surveys will be documented and fed back to residents in a face-to-face meeting. The tutor of each resident will have a face to face talking with the resident in less than six months interval and fill the feedback report. Program director would have comprehensive feedback to each resident. The rotation of certain subspecialty can be extended if residents failed to meet the minimum criteria.

Appendix 1 Table 2 CanMEDS Competency Assessment

Competency	Assessment Method	Frequency
**Medical Expert**		

Knowledge	Written examination （MCQ, short answer questions）<br>Structural case interview	once per 2~4months<br>once per 3 months
Skills	Mini-CEX<br>DOPS<br>SOAP case report<br>360 degree assessment<br>Skill tests<br>HPS simulation（ward practice）	1~2 times per 2~3 months<br>1~2 times per 2~3 months<br>At least once per week<br>once per 2~3 months<br>once per 2~3 months<br>1~2 times per year
**Communicator**		
Knowledge	Structural case interview	Once per 3 months
Verbal skills	Mini-CEX<br>DOPS<br>SOAP case report<br>360-degree assessment	1~2 times per 2~3 months<br>1~2 times per 2~3 months<br>At least once per week<br>once per 2-3 months
Written skills	Medical documents (medical records writing, consultation list, discharge summaries, check the application form)	2 times per 2-3 months
**Collaborator**		
Knowledge	Structural case interview	once per 2-3 months
Skills	DOPS<br>SOAP case report<br>Mentors report<br>Team based teaching<br>Simulation training and feedback	1~2 times per 2~3 months<br>At least once per week<br>Once per 6 months<br>1~2 times per 2 weeks<br>1~2 times per year
Relationships with health care professionals	Nurse/Peer surveys (360-degree assessment)	Once per rotation unit
**Leader**		
Knowledge	Structural case interview	once per 3 months
Management skills	Mini-CEX<br>Nurse/Peer surveys -network based (360-degree assessment)<br>Team based teaching<br>Journal club—solve the problem<br>SOAP case report	1~2 times per 2~3 months<br>Once per rotation unit<br>1~2 times per 2 weeks<br>Once per month<br>At least once per week
**Health Advocate**		
Knowledge	Structural case interview	once per 3 months

Skills	Mini-CEX<br>DOPS<br>360-degree assessment<br>SOAP case report	1~2 times per 2~3 months<br>1~2 times per 2~3 months<br>Once per rotation unit<br>At least once per week
**Scholar**		
Knowledge	Structural case interview<br>short answer questions	once per 3 months<br>once per 3 months
Skills	Journal club<br>360-degree assessment<br>Team teaching（literature reading）	Once per month<br>Once per rotation unit<br>Once per month
Teaching abilities	Turn over the duty<br>360-degree assessment<br>Evaluations from the interns	Every day<br>Once per rotation unit<br>Once per 6 weeks
**Professional**		
Knowledge	Structural case interview	once per 3 months
Skills	Mini-CEX<br>DOPS<br>SOAP case report	1~2 times per 2~3 months<br>1~2 times per 2~3 months<br>At least once per week
Behaviours/Attitudes	360-degree assessment	Once per rotation unit

MCQ: Multiple choice question

DOPS: Direct observation of procedural skills

Mini-CEX: Mini clinical evaluation exercise

HPS: human patient simulation

SOAP: Subjective, Objective, Assessment and Plan
