# Supplementary material for: Exploration of validity evidence for core residency entrustable professional activities in Chinese pediatric residency
Source: Front Med (Lausanne). 2024 Jan 8;10:1301356. doi: 10.3389/fmed.2023.1301356 (PMC10801054; doi:10.3389/fmed.2023.1301356)
Supplement: Supplementary file 1 [file Data_Sheet_1.zip › Data_Sheet_1/Appendix_2.pdf]

Appendix 2

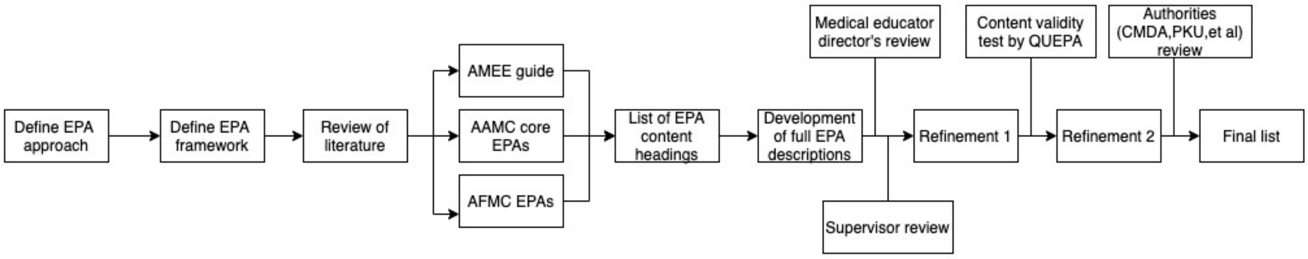

Figure A1 Modified Delphi method process to develop CR-EPAs. CR-EPA: Core Residency EPAs, AMEE: Association for Medical Education in Europe; AAMC: Association of American Medical Colleges; AFMC: Faculties of Medicine of Canada; QUEPA: Quality of Entrustable Professional Activities

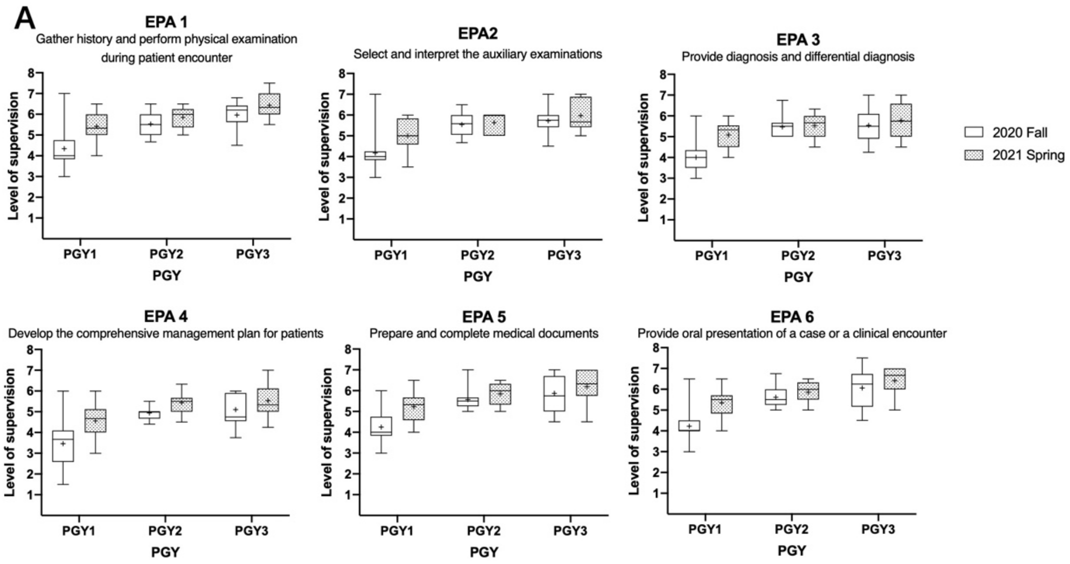

Figure A2 Progression of supervision level by postgraduate year. The dark line and plus mark indicate the median and average respectively, while the box and error bars show, respectively the 25th and 75th and 5th and 95th percentiles. Absence of the box or error bars indicates the same value for the percentiles.

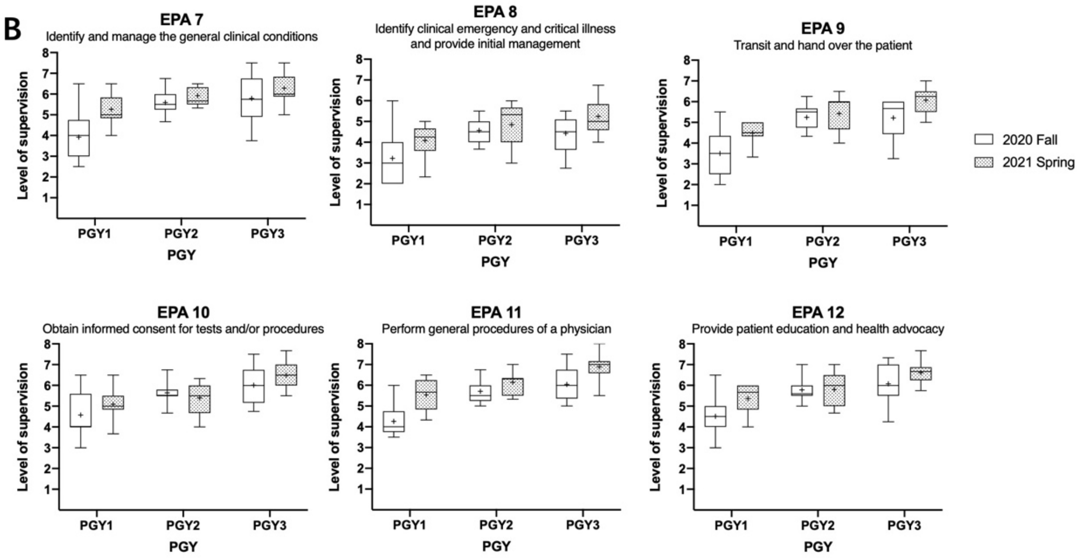

Figure A2 (continued)

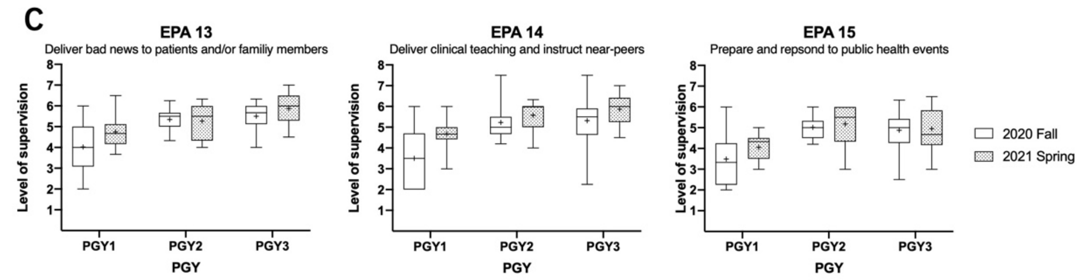

Figure A2 (continued)

Table A1. Correlation coefficients of inter-EPA using Spearman approach

	EPA1	EPA2	EPA3	EPA4	EPA5	EPA6	EPA7	EPA8	EPA9	EPA10	EPA11	EPA12	EPA13	EPA14	EPA15
EPA1	-	0.855	0.819	0.757	0.845	0.898	0.875	0.695	0.764	0.728	0.847	0.77	0.672	0.69	0.646
EPA2	0.855	-	0.926	0.871	0.861	0.859	0.896	0.715	0.664	0.648	0.759	0.716	0.67	0.691	0.761
EPA3	0.819	0.926	-	0.888	0.866	0.848	0.882	0.756	0.704	0.7	0.759	0.701	0.691	0.752	0.804
EPA4	0.757	0.871	0.888	-	0.823	0.824	0.856	0.815	0.734	0.658	0.73	0.69	0.754	0.784	0.777
EPA5	0.845	0.861	0.866	0.823	-	0.92	0.879	0.689	0.757	0.768	0.865	0.765	0.663	0.677	0.631
EPA6	0.898	0.859	0.848	0.824	0.92	-	0.872	0.722	0.791	0.766	0.879	0.799	0.689	0.694	0.645
EPA7	0.875	0.896	0.882	0.856	0.879	0.872	-	0.735	0.757	0.71	0.819	0.72	0.698	0.743	0.705
EPA8	0.695	0.715	0.756	0.815	0.689	0.722	0.735	-	0.782	0.686	0.655	0.696	0.851	0.845	0.832
EPA9	0.764	0.664	0.704	0.734	0.757	0.791	0.757	0.782	-	0.827	0.794	0.819	0.803	0.79	0.681
EPA10	0.728	0.648	0.7	0.658	0.768	0.766	0.71	0.686	0.827	-	0.75	0.883	0.812	0.716	0.646
EPA11	0.847	0.759	0.759	0.73	0.865	0.879	0.819	0.655	0.794	0.75	-	0.786	0.647	0.7	0.541
EPA12	0.77	0.716	0.701	0.69	0.765	0.799	0.72	0.696	0.819	0.883	0.786	-	0.803	0.722	0.644
EPA13	0.672	0.67	0.691	0.754	0.663	0.689	0.698	0.851	0.803	0.812	0.647	0.803	-	0.853	0.797
EPA14	0.69	0.691	0.752	0.784	0.677	0.694	0.743	0.845	0.79	0.716	0.7	0.722	0.853	-	0.826
EPA15	0.646	0.761	0.804	0.777	0.631	0.645	0.705	0.832	0.681	0.646	0.541	0.644	0.797	0.826	-
