# Supplementary material for: Exploration of validity evidence for core residency entrustable professional activities in Chinese pediatric residency
Source: Front Med (Lausanne). 2024 Jan 8;10:1301356. doi: 10.3389/fmed.2023.1301356 (PMC10801054; doi:10.3389/fmed.2023.1301356)
Supplement: Supplementary file 1 [file Data_Sheet_1.zip › Data_Sheet_1/Appendix_3.pdf]

Appendix 3

Entrustable Professional Activities Rating Form for Residents

Instructions:

1. Enstrustable Professional Activities (EPAs) are the degree of entrustment made by the clinical instructors of residents based on daily clinical performance, to make judgement regarding the responsibilities they would take. EPAs could facilitate the translation of clinical competence to clinical practice, to achieve competence-based medical education.
2. This is an onymous evaluation form, with the aim to examine the utility of the EPAs. The results of such examination will only be used research purposes and will be kept confidential. Such results will not be associated with the official performance evaluation of residents that goes into personal documentation.
3. Based Prior Experience, 15 EPAs have been set up by the research team. Please mark the level of entrustment for each EPA based on actual observation, and the verbal descriptions. Comments are more than welcome regarding possible changes towards each EPA.

1. Resident Name

____

2. **EPA1:** Gather history and perform physical examination during patient encounter.
Descriptor: Respect the patient and protect personal confidentiality; Take history with a patient-centered approach; perform comprehensive or focused physical examination based on actual clinical situation.
○ The resident cannot perform the activity at all
○ The resident can achieve the activity with my hands-one all the time
○ The resident can achieve the activity with my full supervision and instruction
○ The resident can achieve the activity mostly alone with my full onsite supervision and double-checking all issues when in need
○ The resident can achieve the activity mostly alone with my occasional onsite supervision and double-checking important issues
○ The resident can achieve the activity alone with my occasional remote supervision and double-check
○ The resident can achieve the activity alone and without my supervision at all
○ The resident can supervise others on this activity
○ Unable to judge

3. **EPA2:** Select and interpret the auxiliary examinations.
Descriptor: Describe the meaning, indications and contradictions of the auxiliary examinations; select reasonable examinations, after considering cost-effectiveness and patient's will; accurately interpret the examination results, and critical values; make reasonable judgements based on clinical situations.
○ The resident cannot perform the activity at all
○ The resident can achieve the activity with my hands-one all the time
○ The resident can achieve the activity with my full supervision and instruction
○ The resident can achieve the activity mostly alone with my full onsite supervision and double-checking all issues when in need
○ The resident can achieve the activity mostly alone with my occasional onsite supervision and double-checking important issues
○ The resident can achieve the activity alone with my occasional remote supervision and double-check
○ The resident can achieve the activity alone and without my supervision at all
○ The resident can supervise others on this activity
○ Unable to rate

4. **EPA3:** Provide diagnosis and differential diagnosis.
Descriptor: Summarize the characteristics of the case; make comprehensive diagnosis; list reasonable differential diagnosis.
○ The resident cannot perform the activity at all
○ The resident can achieve the activity with my hands-one all the time
○ The resident can achieve the activity with my full supervision and instruction
○ The resident can achieve the activity mostly alone with my full onsite supervision and double-checking all issues when in need
○ The resident can achieve the activity mostly alone with my occasional onsite supervision and double-checking important issues
○ The resident can achieve the activity alone with my occasional remote supervision and double-check
○ The resident can achieve the activity alone and without my supervision at all
○ The resident can supervise others on this activity
○ Unable to judge

5. **EPA4:** Develop the comprehensive management plan for patients.
Descriptor: Form reasonable initial management plan based on clinical information and evidence; report to supervisors and modify plan accordingly; communicate the plan with care team and patients to achieve agreement; execute the plan among the care team and perform continuous assessment, modify the plan when needed.
○ The resident cannot perform the activity at all
○ The resident can achieve the activity with my hands-one all the time
○ The resident can achieve the activity with my full supervision and instruction

○ The resident can achieve the activity mostly alone with my full onsite supervision and double-checking all issues when in need
○ The resident can achieve the activity mostly alone with my occasional onsite supervision and double-checking important issues
○ The resident can achieve the activity alone with my occasional remote supervision and double-check
○ The resident can achieve the activity alone and without my supervision at all
○ The resident can supervise others on this activity
○ Unable to judge

6. **EPA5:** Prepare and complete medical documents.
Descriptor: Provide standardized, accurate and complete documentations, with reasonable information presented and in a focused manner; complete the documentation in time.
○ The resident cannot perform the activity at all
○ The resident can achieve the activity with my hands-one all the time
○ The resident can achieve the activity with my full supervision and instruction
○ The resident can achieve the activity mostly alone with my full onsite supervision and double-checking all issues when in need
○ The resident can achieve the activity mostly alone with my occasional onsite supervision and double-checking important issues
○ The resident can achieve the activity alone with my occasional remote supervision and double-check
○ The resident can achieve the activity alone and without my supervision at all
○ The resident can supervise others on this activity
○ Unable to judge

7. **EPA6:** Provide oral presentation of a case or a clinical encounter.
Descriptor: Provide accurate information in a focused and organized manner; report in structured approach with the subject-object-analysis-plan (SOAP) framework; identify room for improvement regarding the presentation and acknowledge clinical uncertainty; introduce patient's understanding and will regarding the condition during reporting.
○ The resident cannot perform the activity at all
○ The resident can achieve the activity with my hands-one all the time
○ The resident can achieve the activity with my full supervision and instruction
○ The resident can achieve the activity mostly alone with my full onsite supervision and double-checking all issues when in need
○ The resident can achieve the activity mostly alone with my occasional onsite supervision and double-checking important issues
○ The resident can achieve the activity alone with my occasional remote supervision and double-check
○ The resident can achieve the activity alone and without my supervision at all
○ The resident can supervise others on this activity
○ Unable to judge

8. **EPA7:** Identify and manage the general clinical conditions.
Descriptor: Identify unusual and emergent clinical findings; prioritize issues based on its urgency; manage the condition appropriately and utilize resources in a reasonable manner; identify personal limitation and ask for help when needed; acknowledge the will of the patients, and continuously reflect on performance and list out directions for improvement.
○ The resident cannot perform the activity at all
○ The resident can achieve the activity with my hands-one all the time
○ The resident can achieve the activity with my full supervision and instruction
○ The resident can achieve the activity mostly alone with my full onsite supervision and double-checking all issues when in need
○ The resident can achieve the activity mostly alone with my occasional onsite supervision and double-checking important issues
○ The resident can achieve the activity alone with my occasional remote supervision and double-check
○ The resident can achieve the activity alone and without my supervision at all
○ The resident can supervise others on this activity
○ Unable to judge

9. **EPA8:** Identify clinical emergency and critical illness and provide initial management.
Descriptor: Identify acute and urgent conditions in a timely and accurate manner and make initial diagnosis; prioritize the order of patient management based on the critical level; seek help from others in time; reasonably allocate and make full use of the available resource, initiate teamwork; respect the will of the patient, communicate with patients in time.
○ The resident cannot perform the activity at all
○ The resident can achieve the activity with my hands-one all the time
○ The resident can achieve the activity with my full supervision and instruction
○ The resident can achieve the activity mostly alone with my full onsite supervision and double-checking all issues when in need
○ The resident can achieve the activity mostly alone with my occasional onsite supervision and double-checking important issues
○ The resident can achieve the activity alone with my occasional remote supervision and double-check
○ The resident can achieve the activity alone and without my supervision at all
○ The resident can supervise others on this activity
○ Unable to judge

10. **EPA9:** Transit and hand over the patient.
Descriptor: Evaluate the risk of patient's transportation based on his/her condition; perform comprehensive communication; prepare and select required medicine, equipment and transportation tools; hand over information accurately and in a structured manner,

make sure the handover is focused and complete; protect the privacy of the patient; demonstrate clear responsibility and effective collaboration.
○ The resident cannot perform the activity at all
○ The resident can achieve the activity with my hands-one all the time
○ The resident can achieve the activity with my full supervision and instruction
○ The resident can achieve the activity mostly alone with my full onsite supervision and double-checking all issues when in need
○ The resident can achieve the activity mostly alone with my occasional onsite supervision and double-checking important issues
○ The resident can achieve the activity alone with my occasional remote supervision and double-check
○ The resident can achieve the activity alone and without my supervision at all
○ The resident can supervise others on this activity
○ Unable to judge

11. **EPA10:** Obtain informed consent for tests and/or procedures.
Descriptor: Clearly introduce the condition to the patient/family; accurately explain the purpose and approach of relevant procedures and potential risk and benefit; provide alternative plans for management; initiate bi-directional communication and make collaborative decisions with patient/family.
○ The resident cannot perform the activity at all
○ The resident can achieve the activity with my hands-one all the time
○ The resident can achieve the activity with my full supervision and instruction
○ The resident can achieve the activity mostly alone with my full onsite supervision and double-checking all issues when in need
○ The resident can achieve the activity mostly alone with my occasional onsite supervision and double-checking important issues
○ The resident can achieve the activity alone with my occasional remote supervision and double-check
○ The resident can achieve the activity alone and without my supervision at all
○ The resident can supervise others on this activity
○ Unable to judge

12. **EPA11:** Perform general procedures of a physician.
Descriptor: Obtain consent before procedure and make required preparation; perform technical procedures; observe patient’s reaction during procedures and make relevant procedures to show compassion towards the patient; identify and manage complications associated with procedures.
○ The resident cannot perform the activity at all
○ The resident can achieve the activity with my hands-one all the time
○ The resident can achieve the activity with my full supervision and instruction
○ The resident can achieve the activity mostly alone with my full onsite supervision and double-checking all issues when in need

○ The resident can achieve the activity mostly alone with my occasional onsite supervision and double-checking important issues
○ The resident can achieve the activity alone with my occasional remote supervision and double-check
○ The resident can achieve the activity alone and without my supervision at all
○ The resident can supervise others on this activity
○ Unable to judge

13. **EPA12:** Provide patient education and health advocacy.
Descriptor: Attend to disease-related health factors; actively initiate general or individualized health advocation; actively respond to health consultation from the patients.
○ The resident cannot perform the activity at all
○ The resident can achieve the activity with my hands-one all the time
○ The resident can achieve the activity with my full supervision and instruction
○ The resident can achieve the activity mostly alone with my full onsite supervision and double-checking all issues when in need
○ The resident can achieve the activity mostly alone with my occasional onsite supervision and double-checking important issues
○ The resident can achieve the activity alone with my occasional remote supervision and double-check
○ The resident can achieve the activity alone and without my supervision at all
○ The resident can supervise others on this activity
○ Unable to judge

14. **EPA13:** Deliver bad news to patients and/or family members.
Descriptor: Make relevant preparation before delivering the bad news; deliver the bad news accurately and appropriately, and in a step-wise manner; show empathy towards the patients, respect and comfort the patient, protect the privacy of the patient; respond appropriately towards the concern and feedback from the patients; confirm with the patients regarding their understanding of the condition; support and collaborate in team, seek help when needed.
○ The resident cannot perform the activity at all
○ The resident can achieve the activity with my hands-one all the time
○ The resident can achieve the activity with my full supervision and instruction
○ The resident can achieve the activity mostly alone with my full onsite supervision and double-checking all issues when in need
○ The resident can achieve the activity mostly alone with my occasional onsite supervision and double-checking important issues
○ The resident can achieve the activity alone with my occasional remote supervision and double-check
○ The resident can achieve the activity alone and without my supervision at all
○ The resident can supervise others on this activity
○ Unable to judge

15. **EPA14:** Deliver clinical teaching and instruct near-peers.
Descriptor: Instruct junior physicians and near-peers according to clinical practice; provide assessment and feedback to junior physicians and near-peers during clinical practice.
○ The resident cannot perform the activity at all
○ The resident can achieve the activity with my hands-one all the time
○ The resident can achieve the activity with my full supervision and instruction
○ The resident can achieve the activity mostly alone with my full onsite supervision and double-checking all issues when in need
○ The resident can achieve the activity mostly alone with my occasional onsite supervision and double-checking important issues
○ The resident can achieve the activity alone with my occasional remote supervision and double-check
○ The resident can achieve the activity alone and without my supervision at all
○ The resident can supervise others on this activity
○ Unable to judge

15. **EPA15:** Prepare and respond to public health events.
Descriptor: Stay cautious and identify to potential public health events; report public health events according to formal procedures and requirements; reasonably respond to public health events and seek help.
○ The resident cannot perform the activity at all
○ The resident can achieve the activity with my hands-one all the time
○ The resident can achieve the activity with my full supervision and instruction
○ The resident can achieve the activity mostly alone with my full onsite supervision and double-checking all issues when in need
○ The resident can achieve the activity mostly alone with my occasional onsite supervision and double-checking important issues
○ The resident can achieve the activity alone with my occasional remote supervision and double-check
○ The resident can achieve the activity alone and without my supervision at all
○ The resident can supervise others on this activity
○ Unable to judge

17. Comments on the overall performance of the resident, including the strength and shortcomings

____

18. Comments on the CR-EPAs rating

____

19. The name of the clinical instructor performing the rating.

____
